# Mineral composition variation in *Boletales* mushrooms—indication of soil properties and taxonomic influence

**DOI:** 10.1007/s11356-024-33916-4

**Published:** 2024-06-07

**Authors:** Przemysław Niedzielski, Marek Siwulski, Małgorzata Szostek, Anna Budka, Sylwia Budzyńska, Magdalena Krzesłowska, Pavel Kalač, Mirosław Mleczek

**Affiliations:** 1 Faculty of Chemistry, Adam Mickiewicz University in Poznań, Uniwersytetu Poznańskiego 8, 61-614 Poznań, Poland; 2 Department of Vegetable Crops, Poznań University of Life Sciences, Dąbrowskiego 159, 60-594 Poznań, Poland; 3 Department of Soil Science, Environmental Chemistry and Hydrology, University of Rzeszów, Zelwerowicza 8B, 35-601 Rzeszów, Poland; 4 Faculty of Environmental and Mechanical Engineering, Department of Construction and Geoengineering, Poznań University of Life Sciences, Wojska Polskiego 28, 60-637 Poznań, Poland; 5 Department of Chemistry, Poznań University of Life Sciences, Wojska Polskiego 75, 60-625 Poznań, Poland; 6 Laboratory of General Botany, Faculty of Biology, Adam Mickiewicz University, Umultowska 89, 61-614 Poznan, Poland; 7 Faculty of Agriculture, Department of Applied Chemistry, University of South Bohemia, 370 04 České Budějovice, Czech Republic

**Keywords:** Major and toxic elements, Species, Substrate characteristics, Wild-growing fungi

## Abstract

The efficiency of element accumulation depends on numerous factors, where the physico-chemical characteristics of the soil seem to be very important, and the role of taxonomic rank in the accumulation of elements by mushrooms seems to be important. The aim of the study was to compare the mineral composition of 7 species belonging to *Leccinum* and *Suillus* genera, collected between 2019 and 2021 from localizations in the west-central part of Poland. The research aimed to indicate the role of selected soil parameters in stimulating/inhibiting the accumulation of elements by selected *Boletales* mushroom species and to answer the question about the role of species belonging to the genus as an indicator determining the specific mineral composition of fruiting bodies. Soil pH and other soil properties (granulometric composition, organic carbon, degree of organic matter decomposition) may significantly affect mushrooms' mineral composition. Mushroom species belonging to *Leccinum* genus exhibited the higher amount of essential major and trace elements than species of *Suillus* genus). It suggests that the affiliation of the studied mushroom species to a specific genus may affect their mineral composition, and the physicochemical properties of the soil may be responsible for the lack of a clear division in the efficiency of element(s) accumulation. Selected species contain high amounts of K, Cu, Fe, and Zn, while others, such as selected *Suillus gravellei* fruiting bodies, also contain As and Cd. The results described serve as an introduction to a broader scientific discussion and require many further studies to confirm the role of taxonomic ranks and the influence of soil characteristics on the accumulation of elements by fruiting bodies.

**Graphical Abstract:**

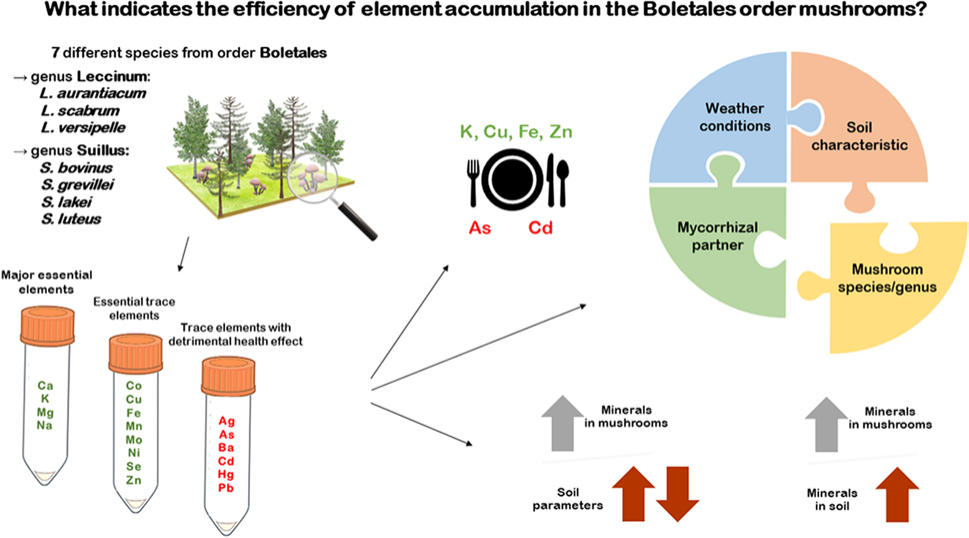

**Supplementary Information:**

The online version contains supplementary material available at 10.1007/s11356-024-33916-4.

## Introduction

According to Geraldi (2021), our knowledge about the diversity of *Boletales* growing in North America, Europe, as well as in East and Southeastern Asia, is extensive. The opposite situation is observed in the case of mushrooms collected from South America, northern Australia, and Sub-Saharan Africa. It is estimated that the *Boletales* lineage emerged in the early Cretaceous period, which may explain the large number of species included in this order (Watkinson and Eastwood 2012). The *Boletales* order comprises 17 families, 96 genera, and 1316 species. The most common families are *Boletaceae* (35 genera, 787 species), *Suillaceae* (3 genera, 54 species), *Gomhidiaceae* (4 genera, 30 species), *Gyroporaceae* (1 genus, 10 species), *Rhizopogonaceae* (3 genera, 152 species), and *Sclerodermataceae* (6 genera, 39 species) (Farid et al. 2021; Kirk et al. 2008). These ectomycorrhizal species are collected from different regions worldwide, especially Eastern Europe, North America, China, and Southern Africa, not limited to those mentioned (Money 2015). Their dynamic development was probably the result of mycorrhizal association with trees, where mutual relationships (transport of metals, phosphorus and nitrogen from mushroom to plant, and carbon compounds from plant to mushroom) are facilitated (Floudas 2021; Lindahl and Tunlid 2014). Due to their sensorial qualities, content of biologically active compounds, or even aroma, especially species of the genus *Boletus*, they have become an important source of vitamins, carbohydrates, proteins, but also selected elements, especially those crucial for human health (Wang et al. 2015).

The analysis of the mineral profile of *Boletales* species has been widely described (Peng et al. 2019; Rizal et al. 2015; Sato and Hattori 2015). Species comparisons were made for those belonging to the same genus or family, as well as among different genera or families (Gałgowska and Pietrzak-Fiećko 2021; Malinowski et al. 2021; Wang et al. 2015). Concurrently with the mineral composition analysis, molecular studies were carried out in the biological diversification of *Boletales* (Binder and Hibbett 2006; Wu et al. 2014). The observed similarities and differences at the molecular level among mushroom species belonging to the *Boletales* order could potentially explain the observed relationships between their mineral profiles. So far, there is no information in the literature describing a close relationship between these two features of mushrooms, creating a niche that necessitates a comprehensive collection of research material from numerous species within a small area to minimize the impact of the environment on the diverse accumulation of individual mushroom species. We could discuss an important ecological indication if such a relationship is confirmed.

The mineral composition of mushrooms is determined by various factors, primarily the characteristics of the ecosystem, with particular emphasis on the content of organic matter and acidity (Dowlati et al. 2021). It should be emphasized that the impact of soil (especially in the organic horizon) as an extremely complex system is significant and is not limited to the key parameters such as geochemistry of the substrate, pH, redox potential, or conductivity (Aloupi et al. 2012; Kokkoris et al. 2019; Nevedrov et al. 2020). The depth and density of the mycelium also play an important role, significantly modifying the accumulation of elements by fruiting bodies (Dowlati et al. 2021). Wang et al. (2015), in their examination of the content of Ca, Cu, Fe, K, Mg, Mn, Na, P, and Zn in ten species of the genus *Boletus*, pointed out differences in the content of these elements in caps and stipes. Despite obtaining valuable results, the authors unfortunately did not indicate statistically significant differences and did not specify the size of the area from which the fruiting bodies were collected. However, the presented characteristics suggest the diverse origins of fruiting bodies from different locations, which may result in the observed differences in the mineral profile of individual species. The crucial role of the fruiting body collection site in modifying the mineral profile was also highlighted by Zhang et al. (2010), researchers of *Boletus edulis* Bull.: Fr. collected from the same site over 2 years. Therefore, the most critical and challenging aspect lies in collecting a relatively representative experimental material from locations with similar physicochemical characteristics. According to Širić et al. (2014), who studied *Boletus reticulatus*, slight variations in the content of selected elements (e.g., Cd, Cr, and Ni), as well as significant differences in the case of others (Fe or Zn), characterize the differential accumulation of elements by a single species. The differentiation may pose a considerable challenge when comparing the mineral composition of multiple species, but it appears to be the only rational and feasible approach to indicate similarities and differences. Wild mushrooms are found only in specific places, and it seems impossible to restrict oneself to a small area with highly homogenous soil characteristics.

The aim of this study was to analyze the mineral composition of seven mycorrhizal mushroom species belonging to *Leccinum* and *Suillus* genera, collected from relatively small areas in the west-central part of Poland between 2019 and 2021. The comparison of mineral profiles was aimed to indicate the potential role of the genus in modulating the efficiency of element accumulation in the studied mushroom species or highlight the significant role of soil as a factor hindering the inference of the higher taxonomic rank's role.

## Materials and methods

### Experimental material

Experimental materials were seven mycorrhizal mushroom species of *Boletales* order (Table 1) belonging to *Boletaceae* (genus *Leccinum*) and *Suillaceae* (genus *Suillus*) families. Genus *Leccinum* was characterized by *Leccinum aurantiacum* (Bull.) Gray (*S*_1_), *Leccinum scabrum* (Bull.) Gray (*S*_2_), and *Leccinum versipelle* (Fr. & Hök) Snell (*S*_3_), while genus Suillus by the following species: *Suillus bovinus* (L.) Roussel (*S*_4_), *Suillus grevillei* (Klotzsch) Singer (*S*_5_), *Suillus lakei* (Murrill) A.H. Sm. & Thiers (*S*_6_), and *Suillus luteus* (L.) Roussel (*S*_7_).

**Table 1:** Characteristics of studied mycorrhizal wild growing mushroom species of *Boletales* order

Mushroom species		Genus	Family	Edibility	Mycorrhizal partner	Number of samples
*S*_1_	*Leccinum aurantiacum* (Bull.) Gray	*Leccinum*	Boletaceae	Edible	*Populus tremula* L	13
S_2_	*Leccinum scabrum* (Bull.) Gray	*Leccinum*	Boletaceae	Edible	*Betula pendula* Roth	18
*S*_3_	*Leccinum versipelle* (Fr. & Hök) Snell	*Leccinum*	Boletaceae	Edible	*Betula pendula* Roth	12
*S*_4_	*Suillus bovinus* (L.) Roussel	*Suillus*	Suillaceae	Edible	*Pinus sylvestris* L	9
*S*_5_	*Suillus grevillei* (Klotzsch) Singer	*Suillus*	Suillaceae	Edible	*Larix decidua* Mill	13
*S*_6_	*Suillus lakei* (Murrill) A.H. Sm. & Thiers	*Suilllus*	Suillaceae	Edible	*Pseudotsuga menziesii* (Mirb.) Franco	5^a^
*S*_7_	*Suillus luteus* (L.) Roussel	*Suillus*	Suillaceae	Edible	*Pinus sylvestris* L	11

^a^Lawn on private property

**Fig. 1:**
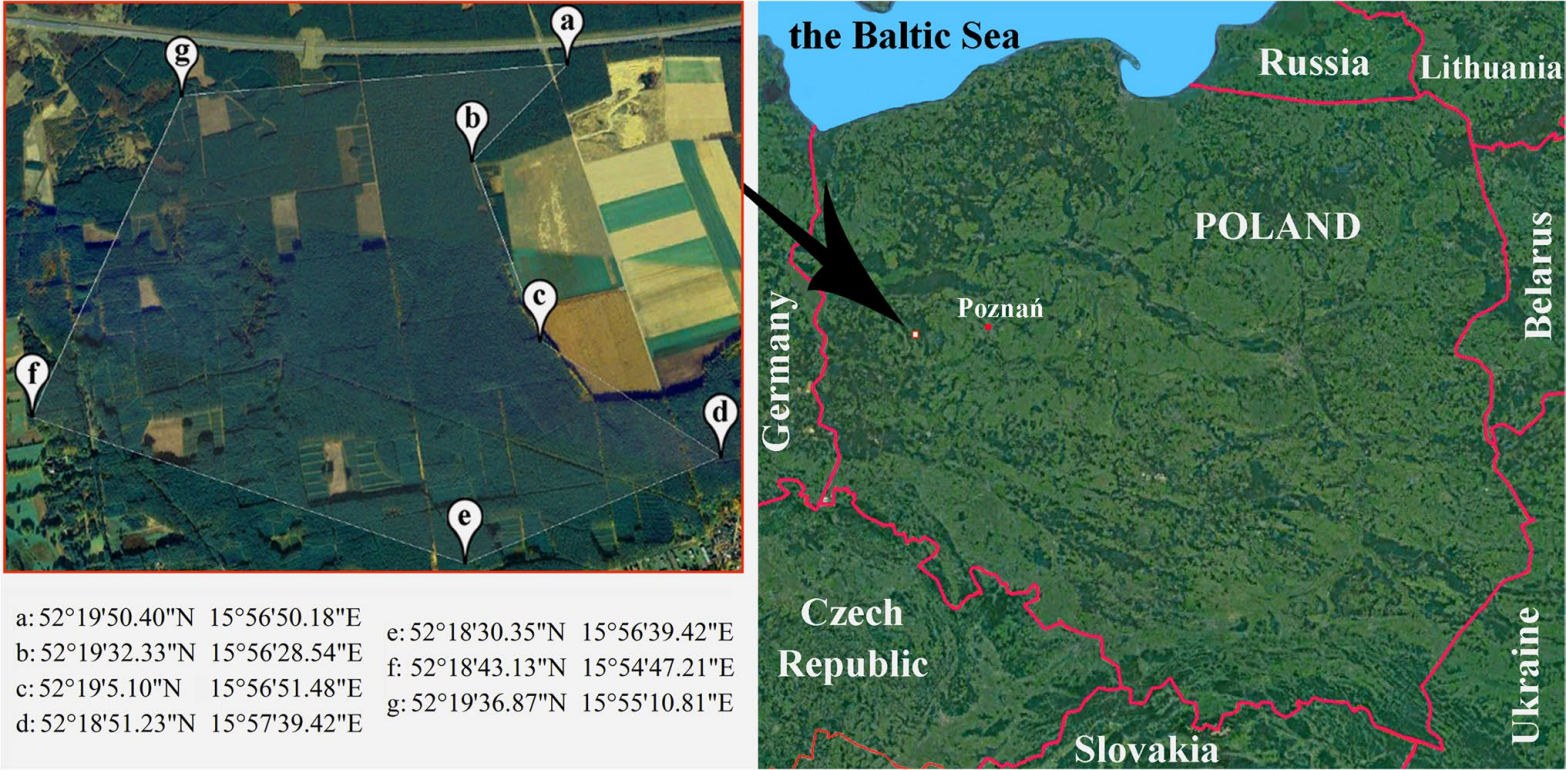
Location of the areas of sample collection. This figure was prepared based on Google Maps

Mentioned species were collected from the forest localized in the west-central part of Poland determined using the GARMIN Oregon® 700 GPS (Garmin, Olathe, USA) (Fig. 1). Mycorrhizal partners of studied mushroom species were *Betula pendula* Roth, *Pinus sylvestris* L., and *Pinus tremula* L. but also *Larix decidua* Mill. In the case of *S*. *lakei*, five fruiting bodies of this species were only collected exclusively in proximity to young *Pseudotsuga menziesii* (Mirb.) Franco growing on the lawn on private property localized in the north part of Poznań (52°24′26″ N, 16°51′37″ E). The choice of the area was deliberate to collect a representative number of fruiting bodies, while for *S*. *lakei*, the only place where five fruiting bodies of this species were located.

### Preparation and analysis of soil samples

For evaluating the selected physicochemical properties, soil samples were taken at a 0–20 cm depth after removing the organic detritus surface layer, as described in an earlier publication (Mleczek et al. 2022). In individual points, soil samples were collected after fruit bodies were harvested from a square of about 10 m^2^ traced. Soil samples were taken at five points of this square (at the four vertices of the square and in the centre) and mixed to obtain representative samples. After being removed from the laboratory, the samples of the soil were air-dried and sieved through a 2 mm sieve. In total, 81 soil samples were analyzed. The particle size distribution was indicated with the laser diffraction method (Laser Particle Sizer ANALYSETTE 22, Fritsch, Idar-Oberstein, Germany). Soil pH values were analyzed in a 1:2.5 substrate-water suspension using a Hanna Instruments (Nusfalaucity, Romania) 4221 pH-meter. Soil organic carbon (SOC) was determined using the Walkley–Black procedure (Nelson et al. 1996), while N was determined using the Kjeldahl method. Additionally, the C:N was calculated as a ratio of soil organic carbon and nitrogen determined in individual soil samples.

### Elements analysis

The content of 18 elements was determined in the collected mushroom species. These elements were divided into the following three groups:

1. Major essential elements (MEEs): calcium (Ca), potassium (K), magnesium (Mg), sodium (Na)
2. Essential trace elements (ETEs): cobalt (Co), copper (Cu), iron (Fe), manganese (Mn), molybdenum (Mo), nickel (Ni), selenium (Se) and zinc (Zn)
3. Trace elements with detrimental health effect (TEWDHE): silver (Ag), arsenic (As), barium (Ba), cadmium (Cd), mercury (Hg), and lead (Pb)

### Procedure

Before analysis, the mushroom and soil samples were dried at 45 ± 5 °C in an electric oven (Thermocenter, Salvislab, Switzerland). In the case of mushroom samples, 0.200–0.300 g (± 0.001) of a dry sample was digested with concentrated nitric acid (HNO_3_, 65%; Sigma-Aldrich, USA) in close Teflon containers in the microwave digestion system Mars 6 Xpress (Mars 6 Xpress, CEM USA). After mineralization, samples were filtered and diluted with deionised water to a total volume of 15.0 mL (MilliQ water purification system, Millipore, Germany).

In the case of soil samples, 0.200–0.300 g (± 0.001) of a dry material was digested with aqua regia solution (2 mL of nitric acid (HNO_3_, 65%; Sigma-Aldrich, USA) and 6 mL of hydrochloric acid (HCl, 30%; Sigma-Aldrich, USA)) in close Teflon containers in the microwave digestion system (Mars 6 Xpress, CEM USA). After that, samples were filtered and then diluted with deionised water to a total volume of 15.0 mL (MilliQ water purification system, Millipore, Germany).

Samples have been diluted 20 times by water just before analysis.

The inductively coupled plasma mass spectrometer PlasmaQuant MS Q (AnalytikJena, Germany) was used to determine the selected elements indicated above. The common conditions were used: Radio Frequency (RF) power 1.35 kW, nebulizer gas flow 1.05 L min^−1^, plasma gas flow 9.0 L min^−1^, auxiliary gas flow 1.5 L min^−1^, signal was measured in 5 replicates (10 scans each). The interferences were reduced with the use of the integrated collision reaction cell (iCRC), which works sequentially in three modes: without gas addition, with helium as collision gas and hydrogen as reaction gas. A reflexION ion mirror was used to adjust the sensitivity for increasing the range of concentrations.

### Analytical method validation

The uncertainty for the analytical procedures was 20%. The detection limits (3-sigma criteria) were indicated with the results (Table S1). The accuracy was checked by analysis of the reference materials: bush branches and leaves (CRM NCSDC 73349); soil (CRM 2709); loess soil (CRM S-1); estuarine sediments (CRM 667 and 405) and the recovery (80–120%) was acceptable for most the elements determined.

For non-certified elements, the recovery in the standard addition method was defined.

### Statistical analysis and calculations

The agricolae, heatmaps, hclust packages (R, Bell Laboratories) were used for all statistical analyses. To compare the mean concentration of 18 elements in soils collected under particular mushroom species and in mushroom species separately, one-dimensional analysis of variance (ANOVA) and Tukey’s HSD (statistically significant difference) test were used. To show similarities between particular mushroom species as regards the content of elements belonging to specific groups of elements (MEEs, ETEs and TEWDHE) and all elements jointly (α = 0.05), the Heatmaps with a cluster analysis were prepared. To assess the degree of linear relationships of the mean concentration of determined elements in soil and fruiting bodies of particular mushroom species, the Pearson linear correlation coefficient (r) values were determined (α = 0.05).

The Daily Intake of Metals/Metalloids (DIM) and the Health Risk Index (HRI) were calculated to present the potential risk for humans better after the collected mushroom intake contained TEWDHE. Values of the Daily Intake of Metals (DIM) were calculated for determined TEWDHE according to the following formula:

$$\text{DIM}= \frac{\text{C metal}/\text{metalloid}\times \text{Dfood intake}}{\text{BW}}$$

where C_metal/metalloid_ is the mean content of metals or metalloid (As) (mg kg^−1^ D.W.), D_food intake_ is the daily mushroom intake (accepted as 250 g of fresh mushrooms = 25 g of dried mushrooms per day (meal)), and BW is regular consumer of 70 kg body weight (Cui et al. 2004; Liu et al. 2015). The Health Risk Index (HRI) was calculated as the ratio of DIM for these elements, and the corresponding values of the appropriate maximum acceptable daily oral dose being RfD_Ag_ = 5, RfD_As_ = 0.3, RfD_Ba_ = 200, RfD_Cd_ = 1.0, RfD_Hg_ = 0.3, and RfD_Pb_ = 3.5 μg kg BW per day, respectively (Barea-Sepúlveda et al. 2022; Sarikurkcu et al. 2020; USEPA 2012). Values of HRI ≤ 0 indicate that the consumption of mushroom fruiting bodies was safe for humans.

## Results

### Physico-chemical characteristics of soils

According to the WRB (2022) classification, the soil from the collection sites of the studied mushroom species was classified into two types: *Albik Brunic Arenosol* (*Dystric*), where the genus *Leccinum* occurs and *Brunic Arenosol* (*Dystric*), where the genus *Suillus* grown (Table 2).

**Table 2:** Physico-chemical characteristics of soils collected under particular mushroom species

Soil	Soil granulometric composition				Soil type (WRB 2022)	pH	SOC	N	C:N
	Sand (2.0–0.05 mm)	Silt (0.05–0.002 mm)	Clay (< 0.002 mm)	Granulometric fraction (WRB 2022)		H_2_O	g kg^−1^	g kg^−1^	
So_1_	73.08^c^ ± 4.05	17.62^a^ ± 3.62	9.39^b^ ± 1.98	SL	*Albik Brunic Arenosol (Dystric)*	4.81^b^ ± 0.09	19.45^ cd^ ± 3.06	1.04^ cd^ ± 0.06	18.76^a ^± 3.27
So_2_	82.39^b^ ± 2.40	11.50^bc^ ± 1.82	6.11^c^ ± 1.60	LS	*Albik Brunic Arenosol (Dystric)*	4.52^c ^± 0.07	19.09^d ^± 1.06	1.00^d ^± 0.04	19.15^a^ ± 0.68
So_3_	88.09^a^ ± 1.58	7.82^d^ ± 1.33d	4.09^d^ ± 0.83	S	*Albik Brunic Arenosol (Dystric)*	4.21^d^ ± 0.06	20.91^abc^ ± 0.77	1.10^c ^± 0.04	18.95^a^ ± 0.44
So_4_	87.67^a^ ± 1.41	8.56^d^ ± 1.13d	3.78^d^ ± 1.09	S	*Brunic Arenosol (Dystric)*	4.29^d^ ± 0.07	21.06^abc^ ± 1.02	1.05^ cd^ ± 0.07	20.02^a^ ± 0.87
So_5_	74.54^c^ ± 3.50	14.85^b^ ± 2.76	10.62^ab^ ± 1.45	SL	*Brunic Arenosol (Dystric)*	4.47^c^ ± 0.05	21.82^ab^ ± 1.18	1.16^b^ ± 0.05	18.72^a^ ± 0.83
So_6_	72.40^c^ ± 2.70	15.00^ab^ ± 1.58	12.60^a^ ± 2.07	SL	*Brunic Arenosol (Dystric)*	5.92^a^ ± 0.05	23.52^a^ ± 0.77	1.28^a^ ± 0.05	18.40^a^ ± 0.93
So_7_	85.73^a^ ± 1.95	9.73^ cd^ ± 1.68	4.55^ cd^ ± 1.13	S	*Brunic Arenosol (Dystric)*	4.74^b^ ± 0.07	22.67^ab^ ± 0.75	1.22^ab^ ± 0.04	18.62^a^ ± 0.68
Mean	80.8	12.2	7.03	–		4.61	20.83	1.10	18.97
Min	68.0	5.0	2.0	–		4.12	10.1	0.94	9.27
Max	91.0	24.0	15.0	–		5.97	24.5	1.34	22.65

Mean ± SD; LS- loamy sand, SL- sandy loam, S- sand, SOC-soil organic carbon. Identical superscripts (a, b) denote non-significant differences between means in columns determined in soils near particular mushroom species according to the post-hoc Tukey’s HSD test

A different particle size composition characterized mineral soil samples (So_1_–So_7_). The mean percentage share of sand, silt, and clay ranged from 73.08–88.09, 7.82–17.62, and 3.78–12.60%, respectively. Three granulometric fractions were distinguished in the analyzed soil samples: silty loam (So_1_, So_5_, So_6_), loamy sand only for So_2_ and sand for the rest places (So_3_, So_4_, So_7_). The pH of the particular soil samples was ranged from 4.21 (So_3_) to 5.92 (So_6_). Most soil samples were acid reactions. Only one soil sample under *S*. *lakei* had a slight acid reaction, significantly higher than that of other soil samples (5.92).

The mean content of SOC was also varied, and in the analyzed soil samples, it ranged from 19.09 to 23.52 g kg^−1^. So_1_ and So_2_ were characterized by the lowest SOC content (19.09 and 19.45 g kg^−1^, respectively), while So_6_ and So_7_ had the highest SOC content (23.52 and 22.67 g kg^−1^, respectively). Similar relationships were also noted regarding nitrogen content. The mean content of this element in the analyzed soil samples ranged from 1.00 to 1.28 g kg^−1^. The lowest SOC content in soils under *L*. *aurantaicum* (So_1_) was observed, while the highest in soil under *S*. *lakei* (So_6_). The C:N ratio in the analyzed soils ranged from 18.4 to 20.02 and did not show significant differences between particular samples.

### Mineral composition of soils

Soil samples collected under studied mushroom species contained similar amounts of Ag, Co, Cu, Mg, Mn, Mo, and Zn despite the relatively wide ranges of determined concentrations amounting respectively: 0.020–0.078, 0.325–1.42, 1.52–10.3, 99.2–400, 68.1–303, 0.010–0.443, and 5.10–27.7 mg kg^−1^ (Tables S2–S4 in Supplementary data). The mean concentration of Ca in studied soils was aligned with the highest values determined in samples under *S*. *lakei* fruit bodies (697 mg kg^−1^) and range of 242–2010 mg kg^−1^ (Table S2). The same was true in the case of K, where the highest concentration of this metal in soils under *L*. *versipelle* was observed (352 mg kg^−1^). The concentration of K in studied soils was included in the range of 98.9–543 mg kg^−1^. The mean concentration of Na was the highest in soils under *L*. *scabrum* and the lowest in soils under *L*. *versipelle* and *S*. *grevillei* (327, 217, and 202 mg kg^−1^, respectively) with the range of 106–502 mg kg^−1^.

The concentration of Fe in soils was included in the range of 1280–3930 mg kg^−1^, with the highest mean value in soils collected under *L*. *aurantiacum* (2800 mg kg^−1^) and the lowest in soils under *L*. *scabrum* and *S*. *luteus* (2480 and 2050 mg kg^−1^, respectively). Soil collected under *S*. *lakei* was characterized by the highest mean concentration of Ni (1.09 mg kg^−1^). In contrast, the lowest values in forest soils under *L*. *aurantiacum* and *L*. *scabrum* were determined (0.681 and 0.579 mg kg^−1^, respectively). The highest mean concentration of Se in soil samples collected under *S*. *lakei* and *S*. *luteus* was determined (0.302 and 0.277 mg kg^−1^, respectively) with lower and similar mean concentrations in soils under the rest of the mushroom species.

The highest mean concentration of As and Cd was determined in soils under *S*. *lakei* (0.533 and 0.465 mg kg^−1^, respectively). These element concentrations ranged from 0.090–0.682 and 0.018–0.542 mg kg^−1^. The highest mean concentration of Ba was determined in soils under *S*. *grevillei*. In contrast, the lowest under *S*. *bovinus* (18.6 and 12.5 mg kg^−1^, respectively) was similar in soils collected under other studied mushroom species. The range of determined concentrations for Ba was from 8.51 to 25.7 mg kg^−1^. The content of Hg concentration in collected soils narrowly ranged from 0.039 to 0.085 mg kg^−1^, and the highest mean concentration in soils under *L*. *versipelle* (0.062 mg kg^−1^) was observed. Similar values were also determined in soils under *L*. *scabrum*, *S*. *grevillei*, and *S*. *luteus* (0.052, 0.051, and 0.052 mg kg^−1^, respectively). Lead was determined in the wide range from 3.95 to 18.8 mg kg^−1^ with the highest mean value in soils under *S*. *bovinus* (13.7 mg kg^−1^) and similar concentrations in soil samples collected under the rest of the species except for *L*. *scabrum*, where the lowest mean concentration of Pb in soil was determined (8.23 mg kg^−1^).

### Mineral composition of mushroom species

#### Major essential elements

The mean content of MEEs was diverse in studied mushroom species (Table 3). The highest mean content of Ca was determined in *L*. *scabrum* (718 mg kg^−1^), while the lowest in *L*. *versipelle*, *S*. *grevillei*, and *S*. *lakei* (217, 252, and 337 mg kg^−1^, respectively). The mean content of Ca in species of genus Suillus was similar, while species of *genus* Leccinum were significantly diverse. The lowest and the highest ranges of Ca were 135 and 850 mg kg^−1^, respectively, for *L*. *versipelle* and *L*. *scabrum*. Fruiting bodies of *L*. *versipelle* were characterized by the highest mean content of K (44,300 mg kg^−1^). This metal was determined in a comprehensive content range for all studied species (from 15,000 to 51,000 mg kg^−1^, respectively, for* L*. *scabrum* and *L*. *versipelle*). Similarly, as for Ca, the mean content determined in species of *Suillus* was similar except for *S*. *luteus* and significantly different in species of genus *Leccinum*.

**Table 3:** Mean content [mg kg^−1^ DW] of major essential elements (MEEs) in selected mushroom species

Mushroom species		Ca	K	Mg	Na
*S*_1_	*Leccinum aurantiacum*	368^bcd^ ± 127	30300^c^ ± 3760	764^b^ ± 142	235^ab^ ± 64.0
		(215–663)	(24,900–36100)	(509–963)	(139–374)
*S*_2_	*Leccinum scabrum*	718^a^ ± 103	22000^d^ ± 3930	418^c^ ± 49.9	207^b^ ± 54.2
		(485–850)	(15,000–30300)	(329–517)	(116–308)
*S*_3_	*Leccinum versipelle*	217^e^ ± 49.2	44300^a^ ± 4550	1020^a^ ± 276	126^d^ ± 19.3
		(135–321)	(35,000–51000)	(743–1550)	(103–178)
*S*_4_	*Suillus bovinus*	369^bcd^ ± 72.7	23500^d^ ± 2610	912^ab^ ± 97.6	270^a^ ± 36.2
		(272–501)	(20,400–27300)	(771–1080)	(212–331)
*S*_5_	*Suillus grevillei*	252^de^ ± 43.8	25500^d^ ± 2890	810^b^ ± 101	52.9^e^ ± 11.6
		(188–320)	(20,100–30800)	(654–1000)	(32.1–70.8)
*S*_6_	*Suillus lakei*	337^b−e^ ± 83.1	25600^ cd^ ± 2540	759^b^ ± 108	128^ cd^ ± 29.0
		(240–429)	(23,400–29600)	(590–856)	(90.6–165)
*S*_7_	*Suillus luteus*	489^b^ ± 83.1	37300^b^ ± 2050	822^b^ ± 158	109^d^ ± 11.2
		(350–593)	(32,100–39700)	(609–1170)	(84.0–126)

Mean ± SD (range); identical superscripts (a, b, c…) denote non-significant differences between means in columns (separately for studied elements) according to the post-hoc Tukey’s HSD test

This tendency was also observed for Mg, where the highest mean content in* L*. *versipelle* fruiting bodies was determined (1020 mg kg^−1^). The mean content of Mg in *L*. *aurantiacum* was lower, while in *L*. *scabrum,* the lowest (764 and 418 mg kg^−1^, respectively) among the studied species of genus Leccinum. The mean content of Mg in species of genus Suillus was almost the same (from 759 to 912 mg kg^−1^, respectively, for *S*. *lakei* and *S*. *bovinus*). The content of Mg was determined in the wide range from 329 to 1550 mg kg^−1^, respectively, for *L*. *scabrum* and *L*. *versipelle*. The highest mean content of Na in *S*. *bovinus* and *L*. *aurantiacum* was determined (270 and 235 mg kg^−1^, respectively), while the lowest mean content in *S*. *grevillei* fruiting bodies (52.9 mg kg^−1^). The content of Na in studied mushrooms was included in the range from 32.1 to 374 mg kg^−1^, respectively, for *S*. *grevillei* and *L*. *aurantiacum*.

**Fig. 2:**
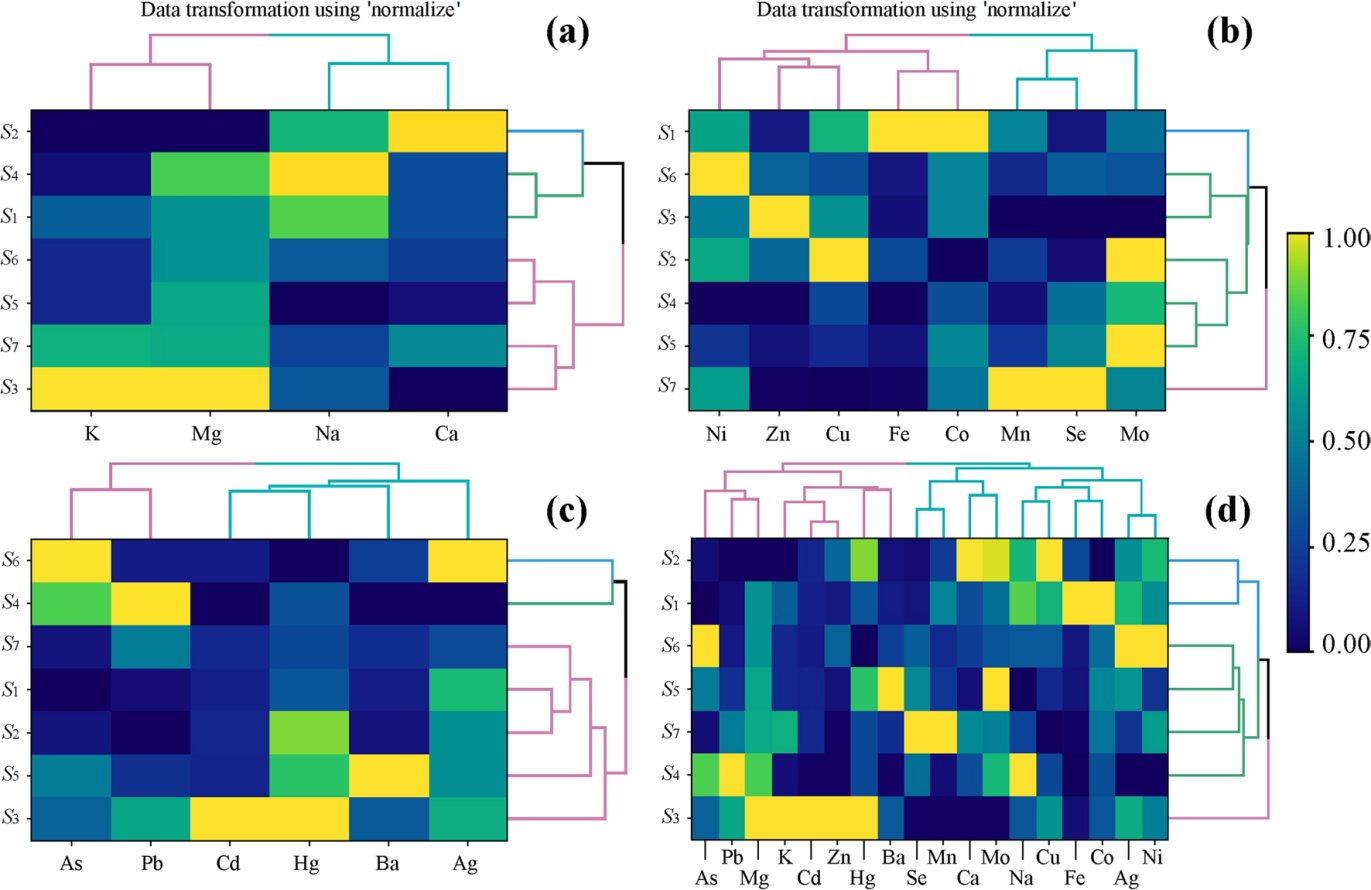
Correlations between the studied mushroom species (S_1_–S_7_) concerning the concentration of MEEs (**a**), ETEs (**b**), TEWDHE (**c**), and all elements jointly (**d**) (heatmap) in mean values with the presentation of a hierarchical tree plot

According to the heatmap prepared for MEEs, K and Mg were similarly accumulated the same as Ca and Na but with different distribution in particular mushroom species (Fig. 2a). Studied mushroom species were divided into two groups: first included *L*. *aurantiacum*, *L*. *scabrum*, and *S*. *bovinus*, while the second composed of the rest studied mushroom species.

#### Essential trace elements

Relationships were observed for Ca, K, and Mg, where differences between species of genus *Leccinum* and the similar mean content of them in species of genus *Suillus* were also visible for ETEs, such as Co, Cu, Fe, Mo, and Zn (Table 4). Fruiting bodies of *L*. *aurantiacum* contained the highest mean amount of Co (0.482 mg kg^−1^), while the lowest mean content was determined in *L*. *scabrum* (0.099 mg kg^−1^). The range of Co in mushrooms was from 0.064 to 0.604 mg kg^−1^, respectively, for *L*. *scabrum* and *L*. *aurantiacum*. *Leccinum aurantiacum* was also characterized by the highest mean content of Fe (212 mg kg^−1^). In contrast, for the rest of the studied species, the mean content of this metal was similar (from 73.2 to 112 mg kg^−1^, respectively, for *S*. *bovinus* and *L. scabrum*). The range of Fe content in studied mushroom species was from 55.8 to 329 mg kg^−1^.

**Table 4:** Mean content [mg kg^−1^ DW] of essential trace elements (ETEs) in selected mushroom species

Mushroom species		Co	Cu	Fe	Mn	Mo	Ni	Se	Zn
*S*_1_	*Leccinum aurantiacum*	0.482^a^ ± 0.069	41.8^b^ ± 7.33	212^a^ ± 71.2	22.9^b^ ± 5.18	0.260^ab^ ± 0.079	1.37^ab^ ± 0.453	0.484^c^ ± 0.098	77.1^bc^ ± 12.5
		(0.347–0.604)	(32.3–58.8)	(104–329)	(17.0–33.2)	(0.184–0.437)	(0.629–1.96)	(0.241–0.605)	(55.8–93.7)
*S*_2_	*Leccinum scabrum*	0.099^c^ ± 0.028	54.2^a^ ± 9.64	112^b^ ± 27.4	17.6^c^ ± 5.15	0.345^a^ ± 0.129	1.40^ab^ ± 0.528	0.449^c^ ± 0.142	102^b^ ± 16.6
		(0.064–0.175)	(43.2–82.8)	(71.2–178)	(12.9–33.5)	(0.194–0.768)	(0.564–2.02)	(0.160–0.609)	(62.6–129)
*S*_3_	*Leccinum versipelle*	0.253^b^ ± 0.035	36.6^bc^ ± 10.2	81.7^b^ ± 13.0	13.4^c^ ± 3.03	0.191^b^ ± 0.092	1.14^bc^ ± 0.237	0.405^c^ ± 0.181	151^a^ ± 56.4
		(0.169–0.304)	(22.4–56.9)	(58.4–100)	(7.56–18.2)	(0.087–0.354)	(0.755–1.43)	(0.207–0.730)	(100–321)
*S*_4_	*Suillus bovinus*	0.218^b^ ± 0.053	24.1^d^ ± 3.31	73.2^b^ ± 10.6	14.5^c^ ± 2.78	0.305^ab^ ± 0.062	0.371^d^ ± 0.089	0.787^b^ ± 0.079	68.5^c^ ± 9.43
		(0.160–0.300)	(17.5–29.0)	(55.8–84.7)	(10.9–19.2)	(0.194–0.380)	(0.201–0.469)	(0.690–0.903)	(52.9–86.5)
*S*_5_	*Suillus grevillei*	0.302^b^ ± 0.099	19.3^de^ ± 4.17	84.4^b^ ± 8.52	17.4^c^ ± 3.85	0.350^a^ ± 0.086	0.685^ cd^ ± 0.213	0.880^b^ ± 0.213	75.0^cb^ ± 12.2
		(0.198–0.471)	(11.0–27.7)	(66.9–101)	(11.3–24.7)	(0.211–0.486)	(0.461–1.13)	(0.466–1.14)	(51.1–91.1)
*S*_6_	*Suillus lakei*	0.260^b^ ± 0.065	25.1^ cd^ ± 4.04	86.6^b^ ± 10.0	16.4^c^ ± 1.19	0.243^ab^ ± 0.061	1.93^a^ ± 0.513	0.733^bc^ ± 0.038	100^cb^ ± 14.3
		(0.189–0.343)	(19.4–29.1)	(78.2–103)	(15.0–18.0)	(0.139–0.304)	(1.06–2.39)	(0.687–0.773)	(82.3–122)
*S*_7_	*Suillus luteus*	0.275^b^ ± 0.069	12.6^e^ ± 3.07	75.5^b^ ± 8.75	31.6^a^ ± 3.36	0.272^ab^ ± 0.065	1.32^ab^ ± 0.343	1.29^a^ ± 0.326	69.3^c^ ± 10.9
		(0.136–0.333)	(7.70–16.0)	(60.0–91.4)	(26.3–39.4)	(0.165–0.406)	(0.782–1.89)	(0.795–1.82)	(50.9–82.8)

Mean ± SD (range); identical superscripts (a, b, c…) denote non-significant differences between means in columns (separately for studied elements) according to the post-hoc Tukey’s HSD test

The mean content of Cu was diverse (from 12.6 to 54.2 mg kg^−1^) in particular mushroom species, with the highest mean content in *L*. *scabrum* fruiting bodies (54.2 mg kg^−1^) and the range from 7.70 to 82.8 mg kg^−1^, respectively for *S*. *luteus* and *L*. *scabrum*. The mean content of Mn was the highest in fruit bodies of *S*. *luteus* and *L*. *aurantiacum* (31.6 and 22.9 mg kg^−1^, respectively) and similar for the rest of the studied mushroom species (from 13.4 to 17.6 mg kg^−1^). The range of determined Mn contents was between 7.56 and 39.4 mg kg^−1^, respectively, for *L*. *versipelle* and *S*. *luteus*. Studied mushrooms were characterized by a similar mean content of Mo ranging from 0.087 to 0.768 mg kg^−1^, respectively, for *L*. *versipelle* and *L*. *scabrum*. A similar mean content of Ni for mushrooms of the genus *Leccinum* was observed.

In contrast, species of the genus *Suillus* were divided into two groups: (1) *S*. *bovinus* and *S*. *grevillei*, and (2) *S*. *lakei* and *S*. *luteus*. The last two species contained the highest amount of Ni in fruiting bodies (1.93 and 1.32 mg kg^−1^, respectively). The content of Ni in mushroom fruiting bodies was determined from 0.201 to 2.39 mg kg^−1^, respectively, for *S*. *bovinus* and *S*. *lakei*. The mean content of Se was similar in most mushroom species of the genus *Leccinum* and usually higher in species of the genus *Suillus*, with the highest content in *S*. *luteus* (1.29 mg kg^−1^). Selenium ranged from 0.160 to 1.82 mg kg^−1^, respectively, for *L*. *scabrum* and *S*. *luteus*. *Leccinum versipelle* was characterized by the highest mean content of Zn (151 mg kg^−1^) and the most comprehensive range (from 100 to 321 mg kg^−1^) of this metal amount. The content of Zn was determined to be in the wide range from 50.9 to 321 mg kg^−1^ (the highest values of Zn and also Co, Cu, and Fe were determined in mushrooms collected near the road).

According to the heatmap prepared for ETE, Mo, Mn, and Se were accumulated similarly in studied mushroom species. At the same time, the rest of the elements created a separate group (Fig. 2b). Comparing ETEs composition in mushroom species, the similarity in content of all these elements in *L*. *versipelle* and *S*. *lakei* with *L*. *scabrum*, *S*. *bovinus*, and *S*. *grevillei* was observed. *Leccinum aurantiacum* and *Suillus luteus* were single separate species characterized by the highest content of Co and Fe (*L*. *aurantiacum*) or Mn and Se (*S*. *luteus*).

#### Trace elements with detrimental health effect

The similar mean content of Ag in mushroom species of genus *Leccinum* was observed, while species of genus *Suillus* were clearly diverse (Table 5). The content of this metal was determined from 0.143 to 1.09 mg kg^−1^, respectively, for *S*. *bovinus* and *L*. *aurantiacum*. The highest mean content of As in *S*. *lakei* and *S. bovinus* (0.812 and 0.707 mg kg^−1^, respectively). Differences in the content of this metalloid in species included in particular genera were observed. The content of As in mushrooms ranged from 0.089 to 1.01 mg kg^−1^, respectively, in *L*. *aurantiacum* and *S*. *lakei*. The highest mean content of Ba was determined in fruiting bodies of *S*. *grevillei* (2.97 mg kg^−1^), while the mean content of this metal in the rest of the species was similar. A high similarity between the studied mushroom species was also observed for Cd, except for *L*. *versipelle*, which is characterized by the highest mean content of this metal (1.98 mg kg^−1^). The content of Cd was determined in the wide range from 0.193 to 2.68 mg kg^−1^, respectively, in *S*. *bovinus* and *L*. *versipelle*. It is worth underlining that the highest content of Cd and the rest of TEWDHE was determined in fruiting bodies collected from the roadside.

**Table 5:** Mean content [mg kg^−1^ DW] of trace elements with detrimental health effect (TEWDHE) in selected mushroom species

Mushroom species		Ag	As	Ba	Cd	Hg	Pb
*S*_1_	*Leccinum aurantiacum*	0.602^a^ ± 0.188	0.258^d^ ± 0.138	1.22^b^ ± 0.363	0.552^b^ ± 0.193	0.329^b^ ± 0.114	0.652^d^ ± 0.148
		(0.404–1.09)	(0.089–0.489)	(0.603–1.83)	(0.301–0.938)	(0.169–0.555)	(0.346–0.846)
*S*_2_	*Leccinum scabrum*	0.522^ab^ ± 0.146	0.304^d^ ± 0.096	1.14^b^ ± 0.344	0.579^b^ ± 0.171	0.614^a^ ± 0.179	0.575^d^ ± 0.149
		(0.248–0.792)	(0.192–0.497)	(0.662–1.86)	(0.344–0.944)	(0.270–0.879)	(0.314–0.759)
*S*_3_	*Leccinum versipelle*	0.579^ab^ ± 0.102	0.466^bc^ ± 0.145	1.68^b^ ± 0.455	1.98^a^ ± 0.349	0.669^a^ ± 0.178	1.39^b^ ± 0.383
		(0.458–0.775)	(0.285–0.734)	(1.08–2.34)	(1.27–2.68)	(0.319–0.952)	(0.810 = 2.02)
*S*_4_	*Suillus bovinus*	0.257^c^ ± 0.082	0.707^a^ ± 0.199	0.985^b^ ± 0.315	0.334^b^ ± 0.190	0.321^b^ ± 0.092	1.84^a^ ± 0.459
		(0.143–0.375)	(0.422–0.916)	(0.557–1.57)	(0.193–0.776)	(0.180–0.418)	(1.36–2.88)
*S*_5_	*Suillus grevillei*	0.533^ab^ ± 0.223	0.524^b^ ± 0.107	2.97^a^ ± 0.942	0.565^b^ ± 0.166	0.552^a^ ± 0.124	0.818^ cd^ ± 0.220
		(0.278–0.926)	(0.419–0.727)	(1.16–4.54)	(0.207–0.782)	(0.376–0.802)	(0.410–1.12)
*S*_6_	*Suillus lakei*	0.731^a^ ± 0.167	0.812^a^ ± 0.184	1.47^b^ ± 0.379	0.518^b^ ± 0.098	0.159^b^ ± 0.039	0.702^ cd^ ± 0.145
		(0.548–0.950)	(0.570–1.01)	(0.874–1.82)	(0.395–0.652)	(0.121–0.216)	(0.459–0.812)
*S*_7_	*Suillus luteus*	0.390^bc^ ± 0.106	0.307^d^ ± 0.065	1.34^b^ ± 0.310	0.597^b^ ± 0.149	0.299^b^ ± 0.072	1.20^bc^ ± 0.367
		(0.253–0.597)	(0.204–0.440)	(0.808–1.77)	(0.359–0.767)	(0.177–0.419)	(0.674–1.88)

Mean ± SD (range); identical superscripts (a, b, c…) denote non-significant differences between means in columns (separately for studied elements) according to the post-hoc Tukey’s HSD test

The highest mean content of Hg in *L*. *scabrum*, *L*. *versipelle*, and *S*. *grevillei* was determined (0.614, 0.669, and 0.552 mg kg^−1^, respectively), while for the rest of the mushroom species was similar. The results showed a similarity of mean Hg content between species belonging to particular genus with some exceptions (*L*. *aurantiacum* in genus *Leccinum* and *S*. *grevillei* in genus *Suillus*). The content of Hg was determined in the range from 0.121 to 0.952 mg kg^−1^, respectively, in *S*. *lakei* and *L*. *scabrum*. Similar relations for the mean content of Pb were also observed. The highest mean content of Pb was determined in *S*. *bovinus* fruiting bodies (1.84 mg kg^−1^), while for the rest species of genus Suillus, the mean content of Pb was similar. The same is the case of species belonging to genus *Leccinum*, where the highest mean content in *L*. *versipelle* was observed (1.39 mg kg^−1^), with the lower and similar mean content of this metal in *L*. *aurantiacum* and *L*. *scabrum* (0.652 and 0.575 mg kg^−1^, respectively).

A heatmap prepared for TEWDHE allowed to show similarity in As and Pb accumulation in studied mushroom species (Fig. 2c). Moreover, two groups of mushroom species characterized by different compositions of these elements were observed. The first group includes *S*. *bovinus* and *S*. *lakei*, while the second is all the rest of the mushroom species. A heatmap prepared for all determined elements shows the existence of three separate groups of mushroom species (Fig. 2d). The first is composed of all species of genus *Suillus*, the second of *L*. *aurantiacum* and *L*. *scabrum*, while the third includes *L*. *versipelle* only. A clear difference between the last one and both the rest of the *Leccinum* species was the cause of the highest content of Cd, K, Hg, Mg, and Zn in *L*. *versipelle.*

Analyzing the correlation between the mean concentration of elements in soils and their content in mushroom fruiting bodies, significant correlations were found for Cu in *L*. *scabrum* (r = 0.5731), Mo and Zn in *L*. *versipelle* (r = 0.6179 and r = 0.5911, respectively), Cu and Zn in *S*. *bovinus* (r = 0.6903 and r = 0.7588, respectively), and also Cu and Fe in *S*. *luteus* (r = 0.7185 and 0.7363, respectively) only.

## Discussion

### Mineral composition of mushrooms

The relatively common occurrence of species belonging to *Boletaceae* and *Suillaceae* families allows for the real possibility of collecting fruiting bodies in the forests of many European countries. This fact is reflected in numerous valuable studies, where the authors assessed the mineral composition of fruiting bodies of species belonging to these families (Dimitrijevic et al. 2016; Gałgowska and Pietrzak-Fiećko 2020; Golubkina and Mironov 2018). Fruiting bodies collected at different times in various regions with diverse physicochemical properties of the soil were characterized by either similar or significantly different contents of specific elements (Kalač 2019). Among the species studied in this work were the fruiting bodies of *L*. *scabrum*, one of the most frequently studied mushroom species (Świsłowski et al. 2020) and *S*. *lakei* collected from a different location due to its interesting mineral composition and its growth under *Pseudotsuga menziesii* exclusively.

#### Content of major essential elements

Brzezicha-Cirocka et al. (2016) analyzed the mineral composition of seven wild-growing mushroom species, including *L*. *aurantiacum*, *L*. *scabrum*, and *S*. *luteus*. The ranges of determined content in our studies for all MEEs in *L*. *aurantiacum* were narrower and entirely within the ranges described by these authors. In contrast, mean content was lower for Ca, K, and Mg (368 and 580, 30300 and 35000, and also 764 and 1100 mg kg^−1^, respectively) and similar for Na (235 and 230 mg kg^−1^, respectively). Similar relationships were also found for *L*. *versipelle*, where the designated intervals were within the ranges described by Brzezicha-Cirocka et al. (2016). Despite the similar mean content of K in *L*. *versipelle* fruiting bodies (41,000 and 44,300 mg kg^−1^, respectively), the ranges of determined concentrations were differentiated (11,000–250,000 and 35,000–51,000 mg kg^−1^, respectively), which was most likely related to differences in the concentration of this element in the soils. Unfortunately, this paper (Brzezicha—Cirocka 2016) does not include the essential soil characteristics like pH, SOC or granulometric fraction, which makes it unable to a broader discussion but is especially important (Malinowski et al. 2021; Sun et al. 2020).

Nevertheless, the critical role of soil is confirmed by previous studies, where, e.g., a lower concentration of Ca in the soil was associated with a lower mean content of this metal in the fruiting bodies of *L*. *scabrum* compared to the data obtained in this work (Mleczek et al. 2015). High similarity in MEEs content in *S*. *luteus* fruiting bodies was also observed in this and Brzezicha-Cirocka et al. (2016) paper despite examination of whole fruiting bodies or their individual parts (caps and stipes). Malinowski et al. (2021) examined the Ca, K, Mg, Na, and P levels in *L*. *scabrum* collected from three regions in NW Poland. Direct comparison of the results presented in the study by Malinowski et al. (2021) is challenging due to slight variations in sample preparation methods. However, the results described by these authors indicate soil parameter variations, potentially reflected in the content of Metal(loid) and essential element species (MEEs) in mushroom fruiting bodies.

This fact may account for differences in the elemental content of mushroom fruiting bodies from different locations, even when using similar or closely related analytical procedures. While MEE content in fruiting bodies may vary in various studies, the ability of species from the same genus to accumulate specific elements may show significant similarity, as demonstrated in the study by Pająk et al. (2020). They observed similar relations between *S*. *luteus*, *S*. *grevillei*, and *S*. *bovinus* for Ca (*S*. *luteus* > *S*. *bovinus* = *S*. *grevillei*), K (*S*. *luteus* > *S*. *grevillei* = *S*. *bovinus*), and Mg (*S*. *bovinus* = *S*. *luteus* = *S*. *grevillei*).

In the case of Na, our results indicate a significantly higher mean content of this metal in *S*. *bovinus* than in *S*. *luteus*, a finding not described by Pająk et al. (2020). These similarities suggest that species belonging to the same genus may exhibit similar element accumulation, especially in the absence of significant differences in their average soil concentration, or they may accumulate significantly larger quantities, providing evidence of their individual capacities.

#### Content of essential trace elements

Similarly, as for MEEs, for selected ETEs (Cu, Fe, and Mn) in our studies, similar mean content in *L*. *aurantiacum* fruiting bodies was found in Brzezicha-Cirocka et al. (2016) paper. Additionally, the apparent difference in mean Zn content was recorded for fruiting bodies of *L*. *aurantiacum* (77.1 and 112 mg kg^−1^, respectively) and *L*. *versipelle* (151 and 170 mg kg^−1^, respectively). Ranges of determined contents of Cu, Fe, Mn, and Zn in *L. aurantiacum* and *L. versipelle* marked in this paper were included in ranges described by Brzezicha-Cirocka et al. (2016), which show similarity in the mineral profile of these mushrooms collected from 2 locations in significant distance (over 300 km). The mean content of Cu, Fe, and Zn in *S*. *luteus* fruiting bodies present in this study results was lower (12.6, 75.5, and 69.3 mg kg^−1^, respectively) than the mean content described by Brzezicha-Cirocka et al. (2016) (20, 360, and 129 mg kg^−1^, respectively). There is no information about soil characteristics; therefore, the steel mill and the opencast sulfur mine may explain the higher content of Fe and Zn, especially in fruiting bodies, resulting from a higher concentration of both metals in the soil. In comparing the results obtained in this study with those described by Pająk et al. (2020), almost the same relations were observed for the Ni accumulation efficiency of *S*. *luteus*, *S*. *grevillei*, and *S*. *bovinus*. However, no similarities were observed for Cu and Zn.

#### Content of trace elements with detrimental health effect

The similarity described earlier in the content of selected elements in the studies presented in this work concerning the results described by Brzezicha-Cirocka et al. (2016) was also found for Ag and Cd in the case of *L*. *aurantiacum*. On the other hand, the apparent difference in mean content of Cd in fruiting bodies of *L*. *versipelle* was observed (1.98 and 14 mg kg^−1^, respectively) and ranges of this toxic metal content (1.27–2.68 and 0.97–57 mg kg^−1^, respectively). The most likely effect of the apparent difference in mean Cd content was the physicochemical characteristics of the soil, rather than a higher concentration of this metal just in the soil resulting from the presence of the steel mill Huta Stalowa Wola. This thesis can be confirmed by almost identical mean Cd content in fruiting bodies *S*. *luteus* (0.53, 0.58 and 0.597 mg kg^−1^, respectively, in caps and stipes described by Brzezicha-Cirocka et al. (2016) and whole fruiting bodies in our studies).

The highest mean content of As was determined in *S*. *lakei* collected from Poznań city and *S*. *bovinus* from the forest area, which in the case of the first species resulted from the highest concentration of As in the soil (0.533 mg kg^−1^). A similar As concentration in soil collected under *S*. *bovinus* to most soil samples was observed. However, 7 of 9 collected samples were characterized by a lower pH value (4.29), similar to the soils under *L*. *versipelle* (4.22). Probably, the high concentrations of Ba in soils under *S*. *grevillei*, Pb under *S*. *bovinus* or Hg under *L*. *versipelle* supported the effective accumulation of these metals in fruiting bodies (Falandysz et al. 2015).

Similar mean content of Cd in *S*. *luteus*, *S*. *grevillei*, and *S*. *bovinus* described in our studies was not described in Pająk et al.’s (2020) studies. The cause of the differences was probably a similar concentration of this metal in soils analyzed in our studies. Contents of Cd and Pb in our studies were also similar to those described by Mirończuk-Chodakowska et al. (2013), who studied *L*. *aurantiacum* and *L*. *scabrum* collected from the eastern region of Poland.

### Role of soil characteristics to mineral profile of mushrooms within the same genus

The occurrence of various species of fungi in a given type of soil is strictly dependent on many factors, both abiotic and biotic. The growth of fungi depends, in particular, on humidity, temperature, soil pH, and the content of readily available organic matter, which serves as a substrate in the nutrition of these organisms (Kuziemska et al. 2019; Rousk et al. 2009). These factors also determine the regulation of the mobility and availability of some elements and, thus, their accumulation in the fruiting bodies of fungi and other living organisms. Numerous studies indicate that mushroom fruiting bodies, thanks to specialized physiological mechanisms enabling easy uptake of elements from various ecosystems, can accumulate excessive amounts of incredibly toxic heavy metals, and their amount is much higher than that specified for crops, vegetables, or fruits (Kalač and Svoboda 2000; Turkekul et al. 2004; Dimitrijevic 2016). These mechanisms largely depend on environmental factors, including soil properties (pH, redox potential, organic matter content), which regulate the mobility and bioavailability of many elements.

The accumulation of many elements, both essential and toxic, in the fruiting bodies of mushrooms depends directly on soil pH (Rousk et al. 2009). However, research in this area provides much contradictory information (Gast et al. 1988; Malinowski et al. 2021). For example, Turkekul et al. (2004) point out that the mushrooms' trace element concentration is mainly affected by their ecosystem and soil’s acidic and organic matter content. In turn, Alonso et al. (2003), based on the conducted research, concluded that neither the pH of the soil nor the content of organic matter influences the uptake and accumulation of various elements by the fruiting bodies of fungi. These differences are because the mechanisms related to the uptake and accumulation of elements by mushroom fruiting bodies are very complex and challenging to analyze, which may be influenced to the greatest extent by the depth and range of mycelium occurrence. The conducted research also observed specific dependencies of the influence of the pH of the analyzed soils on the accumulation of elements of various groups in the fruiting bodies of the studied mushroom species.

The mean Ca content in the soil under *S*. *lakei* (So_6_) was the highest, probably due to this soil's most elevated pH (5.92), as well as the demand of this species just for Ca. Moreover, this soil contained the highest average content of Mg, significant K, Na and Cu, Ni, Se, Zn, soil organic carbon (SOC), and nitrogen. This soil was also characterized by the highest average clay content (12.6%) and the lowest sand (72.4%), which probably influenced Ni content in this soil. This content strictly depends on the granulometric composition of the soil and is generally higher in heavier textured soils (Kabata-Pendias 2004, 2011). The highest concentration of Ni in the So_6_ soil resulted in a more significant accumulation of this element in the fruiting bodies of *S*. *lakei*, and its average content was the highest among the analyzed mushroom species. The solubility of Ni in soils generally increases with a decrease in pH. However, due to its ability to form connections bounds with organic substances, this element can remain highly mobile in many soils and thus absorbable, even in soils with higher pH values, which may explain obtained dependencies (Kabata-Pendias 2011). Moreover, the increased content of As in *S*. *lakei* may result from the fact that Douglas fir, with which this mushroom forms mycorrhiza, is characterized by a particular accumulation of As (Kabata-Pendias 2011). Despite the highest mean content of Ca, Mg, Cu, Se, and Zn in So_6_ soil, their concentration in the fruiting bodies of *S*. *lakei* generally did not differ significantly from other species in the *Suillus* genus (Table 4).

Some dependencies of the influence of pH on the accumulation of individual elements were also observed in the case of fruiting bodies of *L*. *versipelle* and *S*. *bovinus*, collected on soils characterized by the lowest pH value—4.21 (So_3_) and 4.29 (So_4_), respectively (Table 2). These soils were also characterized by the lowest proportion of clay and silt and the highest proportion of sand compared to the other samples. This may indicate this substrate’s high permeability for water and air and the faster mineralization rate of organic remains reaching the surface of these soils. This may also affect the specificity of the accumulation of elements. The soil under *L*. *versipelle* (So_3_) was characterized by the lowest mean Ca concentration (392 mg kg^−1^) and the highest mean K and Hg concentration (352 and 0.062 mg kg^−1^, respectively). In turn, in the soil under *S*. *bovinus* (So_4_), the lowest mean contents were for Ag, As, Ba, and Mn, while the highest was for Na and Pb (Tables S2–S4). Significant differences were found in both species regarding the accumulation of determined elements. *Leccinnum versipelle* was characterized by the highest K, Mg, Zn, Cd, and Hg mean content and the lowest Ca compared to both *S*. *bovinus* and the other analyzed species (Tables 3, 4, and 5). The K content in the fruiting bodies of *L*. *versipelle* was much higher compared to the other studied species (44,300 mg kg^−1^ DW) and 126 times higher than the concentration in the soil, which may indicate that this species has specialized features for accumulating K, regardless of its content in the substrate and regardless of the soil properties (acidic pH favours the leaching of K and reduces the number of its soluble forms). Generally, the concentration of K in fruiting bodies of all mushroom species was many times higher than in the soil. Likewise, the concentration of Mg was also many times higher in fruiting bodies than in the soils for all analyzed mushroom species, in particular in *S*. *bovinus*, *S*. *grevillei*, and *L*. *aurantiacum,* where the concentration in fruiting bodies was about four times higher than in the soil—independently on the soil traits and the element concentration in the soil. Hence, we suppose that high concentrations of these two MEE elements (K, Mg) were involved in the high demand for mushrooms for these elements (Walker and White 2018).

Similar relationships are indicated by Andronikov et al. (2023), and they also apply to other elements, such as Ag, Cd, P, Rb, S, Se, and Zn (Andronikov et al. 2023). Within the *Leccinum* genus, *L*. *versipelle* was characterized by the highest accumulation of Cd and Hg compared to the other species, which can be directly explained by the lowest soil pH under this species and the direct impact of this parameter on the increased accumulation of the elements as mentioned above. In turn, the least Mn, Ag, As, and Ba were found in the fruiting bodies of *S*. *bovinus*, and the most Co and Pb were found compared to other species of the genus *Suillus* and *Leccinum*. This shows again that this soil's low pH could directly impact the obtained relationships, although not in all cases. *L*. *versipelle* and *S*. *bovinus* growing on soils with similar mean pH (4.22 and 4.29, respectively) and As concentration (0.316 and 0.287 mg kg^−1^, respectively) were determined by the significantly different As content in fruiting bodies (0.469 and 0.717 mg kg^−1^, respectively). Despite the lack of differences in other substrate properties (granulometric composition, organic carbon content), the results obtained are significantly different. Since both of these species form mycorrhiza with other tree species, organic matter may influence the different accumulation of As and other elements. The total organic carbon content and the C:N ratio in both soils did not differ significantly. Nevertheless, the species composition of the forest stand is of fundamental importance in creating soil humus of different quality and, therefore, in different possibilities of binding and releasing elements, which may directly impact the obtained relationships (Hobbie et al. 2007). Also, the highest mean concentration of As was determined in soils collected under *S*. *lakei*, while significantly lower in soils under *S*. *bovinus* (0.533 and 0.287 mg kg^−1^, respectively). In comparison, the mean content in the fruiting bodies of these species was similar and the highest (0.812 and 0.707 mg kg^−1^, respectively). The obtained result can be fully explained by the difference in mean pH values (5.92 and 4.29, respectively).

An apparent effect of the soil pH can also be seen in the case of Cd, whose highest concentration was determined in the soil under *S*. *lakei* and *L*. *versipelle* (0.465 and 0.357 mg kg^−1^, respectively), with almost four times higher content of this metal in fruiting bodies of the last one only. Similar mean concentrations of Se were the highest in soils under *S*. *lakei* and *S*. *luteus* (0.302 and 0.277 mg kg^−1^, respectively), while the mean content of Se was 0.733 and 1.29 mg kg^−1^, respectively), which may be explained by a significant difference in pH of soils (5.92 and 4.74, respectively). On the other hand, the similar mean content of Se in *S*. *bovinus* and *S*. *grevillei* fruiting bodies growing on soil with lower pH values (4.29 and 4.47, respectively) was observed despite this metal’s significantly lower mean concentration in soils.

The described differences, which were undoubtedly caused to some extent by the parameters characterizing the soil, hinder the accurate assessment of the genus’s role in regulating species’ mineral composition. The heatmaps prepared based on obtained results show similarities but also differences between the mineral profile of studied mushroom species belonging to the same genus, which mainly suggests the role of the species as a determinant of the accumulation of a specific element(s) (Fig. 2a–d). A heatmap in Fig. 2a shows the similarity of *S*. *bovinus* to *L*. *aurantiacum* and *L*. *scabrum* and also includes *L*. *versipelle* to the rest of the *Suillus* species.

The described observations do not constitute definitive proof that the belonging of mushroom species to a given genus determines their ability to accumulate higher or lower of the studied elements. On the other hand, this does not exclude such a possibility. What is certain is that the accumulation of elements is a function of many factors, each of which has a specific effect, as exemplified by the results obtained for Se. Similar mean concentrations of this element were the highest in soils under *S*. *lakei* and *S. luteus* (0.302 and 0.277 mg kg^−1^, respectively), while the mean content of Se was 0.733 and 1.29 mg kg^−1^, respectively, which may be explained by a significant difference in pH of soils (5.92 and 4.74, respectively). On the other hand, the similar mean content of Se in *S*. *bovinus* and *S. grevillei* fruiting bodies growing on soil with lower pH values (4.29 and 4.47, respectively) was observed despite this metal's significantly lower mean concentration in soils. Described observations and relationships indicated that any interpretation of the results regarding the content of elements in mushrooms requires considering the characteristics of the substrate from which they were collected. However, demonstrating similarities between species requires many further studies, evaluating the most extensive number of fruiting bodies collected from areas with relatively similar but also significantly different physico-chemical characteristics of soils.

Correlations presented in Table 6 were identified for only four elements (Cu, Fe, Mo, and Zn) and four mushroom species (*L*. *scabrum*, *L*. *versipelle*, *S*. *bovinus*, and *S*. *luteus*). Kuziemska et al. (2019) previously indicated a significant correlation between the concentration of Cu and Zn in soils under *S*. *luteus* and these metals in its mushroom fruiting bodies. In our study, significant correlations for Cu and Fe were observed, similar to the findings by Murati et al. (2022). Both our study and that of Kuziemska et al. (2019) revealed no significant correlations for Ni in *S*. *luteus*, as well as for Ni and Zn in *L*. *scabrum*.

**Table 6:** Correlation between mean concentration of elements determined in soil and their mean content in fruiting bodies of studied mushroom species

Mushroom species	Ca	K	Mg	Na	Co	Cu	Fe	Mn	Mo	Ni	Se	Zn	Ag	As	Ba	Cd	Hg	Pb
*Leccinum aurantiacum*	0.5103	0.3223	0.2897	0.1260	0.0541	0.2497	− 0.3255	0.1419	0.1776	0.2427	0.0933	− 0.4529	0.4666	0.1726	0.3365	0.3160	0.0838	0.4665
*Leccinum scabrum*	− 0.0818	− 0.3058	0.1494	− 0.1043	− 0.0745	0.5731*	0.0764	− 0.3011	0.1836	− 0.1445	0.0478	0.2573	0.0720	0.0478	0.1531	− 0.2415	− 0.3290	0.1566
*Leccinum versipelle*	0.3754	0.4004	0.4098	0.0856	0.1466	0.3202	0.1912	0.2073	0.6179*	0.0443	0.3546	0.5911*	0.2928	0.1588	0.0082	0.2180	0.4781	0.0587
*Suillus bovinus*	0.2863	0.2781	0.4277	0.1648	0.3188	0.6903*	0.0413	0.6184	0.4853	0.6606	− 0.0428	0.7588*	0.4972	0.0935	0.0803	0.1903	0.3661	0.6562
*Suillus grevillei*	0.3492	0.0020	0.5252	0.0091	0.1219	0.2022	0.2502	0.2583	0.0127	0.2017	0.3876	0.0147	0.4914	0.2852	0.0100	0.1957	0.0176	0.1516
*Suillus* *lakei*	0.6533	0.2712	0.3639	0.0626	0.4784	0.2424	0.5437	0.6304	0.2776	0.7282	0.6023	− 0.3289	0.7894	0.3441	0.2993	0.1245	0.5671	0.6091
*Suillus* *luteus*	0.1218	0.1996	0.1216	0.2055	− 0.2593	0.7185*	0.7363*	− 0.2384	0.2227	0.5178	0.5803	0.0562	0.2587	0.4079	0.5011	− 0.0432	0.1134	0.0441

^*^Correlation significant at the 0.05 level

Significant correlations for Cu in *L*. *scabrum*, as indicated by Kuziemska et al. (2019), align with our results. The role of soil element concentrations in modifying the efficiency of their accumulation in fungal fruiting bodies is certain, but definitively pinpointing specific correlations requires further research. Additionally, maintaining cautious optimism is necessary, as evidenced by observations from Wang et al. (2015), who studied ten wild-growing Boletus species with corresponding underlying soils. The authors highlighted correlations between Ca concentration in soil and mushrooms, but significant correlations for Ca in stipes (not in caps) were only observed.

Moreover, identified correlations between soil/caps of mushrooms (e.g., Ca-Fe, Zn-K, K-Mg, and Zn-Mg) or soil/stipes of mushrooms (Cu-Mg, Fe–Mg, Mg-Fe, Zn-Mg, or Zn-Na) suggest the significant role of specific elements in the efficiency of accumulation of others. Furthermore, due to the varied transport of elements within fruiting bodies, these correlations may be either significant or not. It seems justified to state that the correlations we described, although calculated for the elemental content in whole fruiting bodies, do not unequivocally prove the bioindicative capabilities of selected species. However, they strongly indicate that the concentration of elements in soil significantly influences the ability of fungal fruiting bodies to accumulate elements.

### Mineral composition and health implication

According to EFSA (2017), the adequate intake (AI) values defining an adult's daily requirements for Ca, K, Mg, and Na were established at 950, 3500, 350, and 2000 mg, respectively. Due to the permanent increase of mushroom production and intake with a simultaneous reduction of the single meal weight of fruiting bodies, a portion of 200 g of fresh weight, which corresponds to 20 g of dry matter of mushrooms, was used for calculations (Mleczek et al. 2023). Mean contents of Ca, K, Mg, and Na in 20 g portion of *Leccinum* species were: 9.45, 614, 13.8, and 3.86 mg, respectively, while of *Suillus* species: 7.19, 569, 16.6, and 2.61 mg, respectively. These values corresponded to 0.99, 17.6, 3.95, and 0.19% of AI for *Leccinum* and 0.76, 16.3, 4.75, and 0.13% of AI for *Suillus* species. These results indicate that consuming harvested fruiting bodies was associated with providing small amounts of MEEs, except for potassium.

In the case of determined ETEs (Cu, Fe, Mn, Mo, Se, and Zn), the following AI are established: 1.6, 11.0, 3.0, 0.065, 0.07, and 11.7 mg per day. The mean contents of the mentioned elements in 20 g meal of *Leccinum* species were 0.911, 2.67, 0.360, 0.006, 0.009, and 2.16 mg, respectively, while of *Suillus* species: 0.385, 1.59, 0.414, 0.006, 0.019, and 1.50 mg, respectively. These values corresponded to 56.9, 24.3, 12.0, 8.49, 12.8, and 18.5% of AI for *Leccinum* species, respectively and 24.0, 14.4, 13.8, 9.31, 27.4, and 12.8% of AI for *Suillus.* These values indicate that the consumption of the studied fruiting bodies was associated with the supply of a significant amount of Cu and Fe in the case of *Leccinum* species (mainly *L*. *scabrum* and *L*. *aurantiacum*), the same as Se in the case of *Suillus* species (mainly *S*. *luteus*). The mean content of Cu in 20 g portion of *L*. *scabrum* was 1.08 mg (range from 0.863 to 1.66 mg), which was 67.7% of AI_Cu_ (range from 53.9 to 104). In the case of *L*. *aurantiacum*, the mean content of Fe in a single portion was 4.23 mg (range from 2.07 to 6.58 mg), which was 38.5% of AI_Fe_ (range from 18.8 to 59.8%). *Suillus luteus* was characterized by the highest mean content of Se (1.29 mg kg^−1^). The content of Se in 20 g mean prepared of this species was 0.026 mg (range from 0.016 to 0.036 mg), and this corresponded to 36.8% of AI (range from 22.7 to 52.0%). According to EFSA (2015), the Tolerable Daily Intake (TDI) for Ni was established at 2.8 μg kg^−1^ body weight (bw), while body mass for women and men of 58.5 and 68.1 kg, respectively (EFSA NDA Panel 2013), therefore the maximal amount of Ni is 164 and 191 μg per day. Intake of 20 g portion of *Leccinum* species covers 16.1% of the daily demand for this metal by women and 13.8% by men, while for *Suillus* species, these values are 11.7 and 10.0%, respectively. Underlining the mushroom species characterized by the highest Ni content (*L*. *scabrum* and *S. lakei*) satisfied less than 30% of TDI_Ni_, which is worth it.

Generally, the most critical problem in wild-growing mushrooms is the content of toxic elements, which should be as low as possible. According to Commission Regulation (EC 2008), the maximum level of As, Cd, and Pb in foodstuffs such as mushrooms is 0.5, 1.0, and 3.0 mg kg^−1^ DW, respectively. The mean content of As in *Leccinum* and *Suillus* species was 0.335 and 0.542 mg kg^−1^, respectively, corresponding to 67 and 108% of the acceptable level, respectively. It is worth underlining that content higher than 0.5 mg kg^−1^ was determined in 25, 56, and 23% of *L*. *versipelle*, *S*. *bovinus*, and *S. grevillei*, respectively. In the case of *L*. *aurantiacum*, *L*. *scabrum*, and *S*. *luteus*, all studied fruiting bodies contained As below 0.5 mg kg^−1^. In contrast, the opposite situation was observed for all *S. lakei* collected from the city.

Of the examined mushroom species, only the *L*. *versipelle* fruiting bodies contained higher than 1.0 mg kg^−1^ of Cd. Similar contents (range from 0.46 to 5.1 mg kg^−1^) exceeding the norm indicated above for this toxic metal were described for this species, e.g., by Mędyk et al. (2017), while Krejsa et al. (2021) stated the ability of *L*. *versipelle* to effective accumulation of Cd (BCF = 4.7). However, it should be remembered that the higher accumulation of Cd in fruiting bodies is mainly due to a higher concentration of this metal in the soil and a lower pH (Sun et al. 2017), hence the fact that 11 of 12 fruiting bodies of *L*. *versipelle* were collected from the roadside. The mean content of Pb in fruiting bodies of *Leccinum* and *Suillus* were 0.827 and 1.16 mg kg^−1^, respectively, which is under the limit established for this metal. It should be emphasized that none of the values determined for Pb exceeded 3 mg kg^−1^, which indicates that the consumption of fruiting bodies studied in this work was not associated with the risk of excessive absorption of Pb (Jarzyńska and Falandysz 2012; Mleczek et al. 2013).

The presence of Hg in the collected fruiting bodies also requires a comment on this point. According to JECFA (2012), for adults weighing 70 kg, the Provisional Tolerable Weekly Intake (PTWI) was established at 0.28 mg per week. The mean Hg content in 20 g portion of dry fruiting bodies of species belonging to genera *Leccinum* and *Suillus* was 0.011 and 0.007 mg, respectively. At the same time, the highest values were 0.019 and 0.016 mg, respectively. These values indicate that consuming fruiting bodies containing the highest Hg amount, even 7 days a week will fill 47.5 and 40% of PTWI_Hg_. The mean values are 27 and 18.6%, respectively, combined with the fact that consumption of Hg from other food products does not involve a risk to human health and life.

To clearly indicate the risk associated with the consumption of the analyzed mushroom species containing the determined TEWDHE, DIM and HRI values were calculated (Table 7). In the case of DIM, values in Table 7 were lower than the Provisional Tolerable Daily Intake (PTDI) values for As, Cd, and Hg being 2.14, 0.82, and 0.57 μg kg^−1^ BW per day (JECFA 2021), and also the Provisional Tolerable Weekly Intake (PTWI) for Pb being 25 μg kg^−1^ BW per week. Additionally, HRI values were lower than 1, which indicates that intake of studied mushrooms was safe (Leung et al. 2008). However, it should be remembered that the calculated indexes refer to mean content; hence, for the highest As and Cd contents in the fruiting bodies of the previously described mushroom species (*L*. *versipelle*, *S. bovinus*, and *S*. *grevillei*), values exceeding the maximum levels established for them were found.

**Table 7:** Calculated values of daily intakes of metal/metalloid (DIM) [μg kg^−1^ BW per meal] and Health Risk Indexes (HRI) for studied mushroom species

Mushroom species		Index	Ag	As	Ba	Cd	Hg	Pb
*S*_1_	*Leccinum aurantiacum*	DIM	0.215	0.092	0.435	0.197	0.117	0.233
		HRI	0.043	0.308	0.002	0.197	0.391	0.067
*S*_2_	*Leccinum scabrum*	DIM	0.186	0.109	0.406	0.207	0.219	0.205
		HRI	0.037	0.362	0.002	0.207	0.731	0.059
*S*_3_	*Leccinum versipelle*	DIM	0.207	0.167	0.599	0.707	0.239	0.498
		HRI	0.041	0.555	0.003	0.707	0.797	0.142
*S*_4_	*Suillus bovinus*	DIM	0.092	0.253	0.352	0.119	0.115	0.658
		HRI	0.018	0.842	0.002	0.119	0.382	0.188
*S*_5_	*Suillus grevillei*	DIM	0.190	0.187	1.060	0.202	0.197	0.292
		HRI	0.038	0.623	0.005	0.202	0.658	0.083
*S*_6_	*Suillus lakei*	DIM	0.261	0.290	0.524	0.185	0.057	0.251
		HRI	0.052	0.966	0.003	0.185	0.190	0.072
*S*_7_	*Suillus luteus*	DIM	0.139	0.110	0.480	0.213	0.107	0.428
		HRI	0.028	0.366	0.002	0.213	0.356	0.122

## Conclusions

Mushrooms are crucial component of forest ecosystems, and an apparent increase in interest in them drives further research to characterize them even more broadly. The results obtained in this work indicated the content of selected elements in the fruiting bodies of species of the genera *Leccinum* and *Suillus*, similar to many other studies conducted both in Poland and in the world. The identified differences in the mineral composition of fruiting bodies may result from their ability to accumulate specific elements effectively. On the other hand, in many cases, the higher accumulation of elements in fruiting bodies caused by their higher concentration in the soil was explained, as well as the influence of soil characteristics on the mineral profile of mushrooms. These studies included the analysis of only 81 fruiting bodies collected over 3 years. Hence, they cannot provide unambiguous evidence for the role of the genus, especially soil pH, as the primary determinant of the accumulation of elements in mushrooms. It should be emphasized, however, that higher accumulation of the element in the fruiting body resulting from its higher concentration in the soil or lower pH may distort the picture of the real potential of a given mushroom species to accumulate them. Other properties of the substrate, such as granulometric composition and the quality of organic matter, also impact the accumulation of elements. Thus, it is possible that species belonging to the same genus are not found to be similar due to the different accumulation of a specific element or their total content.

The collected fruiting bodies of the genera *Leccinum* and *Suillus* provided optimal amounts of Cu, K, Fe, and Zn. Additionally, it was found that 25, 56, and 23% of *L. versipelle*, *S*. *bovinus*, and *S*. *grevillei* fruiting bodies, respectively, contained a higher amount of As that the maximum accepted level, the same as *L*. *versipelle* fruiting bodies due to the Cd content. This does not mean, however, that these species should not be eaten. However, the fruiting bodies of *L*. *versipelle* were collected from soils with the lowest pH from places on the roadside and growing on soil with a relatively higher concentration of this metal than other soils. It should be remembered that mushrooms for consumption should be collected further away from the road because of the lack of direct impact of road traffic. Still, the chemical characteristics of soils conducive to the accumulation of metals may indicate a risk of acquiring contaminated fruiting bodies.

### Supplementary Information

Below is the link to the electronic supplementary material.

Supplementary file1 (DOCX 26 KB)

## Data Availability

Not applicable.
