# Supplementary material for: Mineral composition variation in Boletales mushrooms—indication of soil properties and taxonomic influence
Source: Environ Sci Pollut Res Int. 2024 Jun 7;31(28):41137–54. doi: 10.1007/s11356-024-33916-4 (PMC11189970; doi:10.1007/s11356-024-33916-4)
Supplement: Supplementary file 1 — Supplementary file1 (DOCX 26 KB) [file 11356_2024_33916_MOESM1_ESM.docx]

Supplementary data to:

"Mineral Composition Variation in Boletales Mushrooms - Indication of Soil Properties and Taxonomic Influence" by Niedzielski et al.

Table S1. Detection limits for elemental analysis by ICP MS

| Element | | DL |
| --- | --- | --- |
| MEEs | Ca | 33.2 |
|  | K | 14.4 |
|  | Mg | 3.26 |
|  | Na | 4.66 |
| ETEs | Co | 0.0065 |
|  | Cu | 0.1230 |
|  | Fe | 4.19 |
|  | Mn | 0.032 |
|  | Mo | 0.0041 |
|  | Ni | 0.0134 |
|  | Se | 0.099 |
|  | Zn | 0.0581 |
| TEWDHE | Ag | 0.018 |
|  | As | 0.005 |
|  | Ba | 0.034 |
|  | Cd | 0.0094 |
|  | Hg | 0.0363 |
|  | Pb | 0.0864 |

Table S2. Mean concentration [mg kg^-1^] of major essential elements in soils under particular mushroom species

| Soil | Ca | K | Mg | Na |
| --- | --- | --- | --- | --- |
| So_1_ | 504^ab^ ± 92.3 | 290^ab^ ± 103 | 197^a^ ± 52.1 | 281^ab^ ± 91.7 |
| So_2_ | 549^ab^ ± 176 | 242^b^ ± 85.5 | 202^a^ ± 65.0 | 327^a^ ± 86.3 |
| So_3_ | 392^b^ ± 83.1 | 352^a^ ± 103 | 265^a^ ± 76.1 | 217^b^ ± 64.8 |
| So_4_ | 522^ab^ ± 139 | 283^ab^ ± 75.8 | 211^a^ ± 74.4 | 265^ab^ ± 68.6 |
| So_5_ | 499^ab^ ± 104 | 234^b^ ± 98.1 | 219^a^ ± 61.3 | 202^b^ ± 50.2 |
| So_6_ | 697^a^ ± 182 | 328^ab^ ± 80.5 | 272^a^ ± 55.5 | 294^ab^ ± 38.2 |
| So_7_ | 588^a^ ± 181 | 249^ab^ ± 84.5 | 256^a^ ± 84.2 | 269^ab^ ± 63.2 |
| Range | 242 - 1010 | 98.9 - 543 | 99.2 - 400 | 106 - 502 |

Mean ± SD; identical superscripts (a, b) denote non-significant differences between means in columns (separately for studied elements) according to the post-hoc Tukey’s HSD test

Table S3. Mean concentration [mg kg^-1^] of essential trace elements in soils under particular mushroom species

| Soil | Co | Cu | Fe | Mn | Mo | Ni | Se | Zn |
| --- | --- | --- | --- | --- | --- | --- | --- | --- |
| So_1_ | 0.856^a^ ± 0.212 | 5.62^a^ ± 1.41 | 2800^a^ ± 646 | 121^a^ ± 46.7 | 0.198^a^ ± 0.114 | 0.681^b^ ± 0.247 | 0.181^b^ ± 0.078 | 12.7^a^ ± 3.66 |
| So_2_ | 0.788^a^ ± 0.239 | 6.07^a^ ± 2.02 | 2480^b^ ± 478 | 147^a^ ± 56.5 | 0.234^a^ ± 0.098 | 0.579^b^ ± 0.148 | 0.172^b^ ± 0.048 | 14.4^a^ ± 4.11 |
| So_3_ | 0.806^a^ ± 0.283 | 5.80^a^ ± 1.93 | 2210^ab^ ± 592 | 119^a^ ± 64.2 | 0.226^a^ ± 0.086 | 0.752^ab^ ± 0.286 | 0.181^b^ ± 0.058 | 15.5^a^ ± 4.19 |
| So_4_ | 0.947^a^ ± 0.264 | 5.37^a^ ± 2.21 | 2180^ab^ ± 493 | 117^a^ ± 52.4 | 0.218^a^ ± 0.103 | 0.820^ab^ ± 0.308 | 0.175^b^ ± 0.046 | 14.0^a^ ± 4.63 |
| So_5_ | 0.843^a^ ± 0.223 | 4.95^a^ ± 1.25 | 2620^ab^ ± 541 | 135^a^ ± 36.3 | 0.245^a^ ± 0.088 | 0.766^ab^ ± 0.265 | 0.162^b^ ± 0.056 | 12.5^a^ ± 4.17 |
| So_6_ | 0.717^a^ ± 0.199 | 6.12^a^ ± 1.56 | 2380^ab^ ± 493 | 146^a^ ± 46.2 | 0.192^a^ ± 0.109 | 1.09^a^ ± 0.253 | 0.302^a^ ± 0.071 | 18.1^a^ ± 6.11 |
| So_7_ | 0.683^a^ ± 0.232 | 4.45^a^ ± 1.42 | 2050^b^ ± 365 | 161^a^ ± 46.8 | 0.209^a^ ± 0.084 | 0.699^ab^ ± 0.160 | 0.277^a^ ± 0.060 | 12.5^a^ ± 4.73 |
| Range | 0.325 – 1.42 | 1.52 – 10.3 | 1283 - 3930 | 68.1 - 303 | 0.010 – 0.443 | 0.233 – 1.57 | 0.111 – 0.458 | 5.10 – 27.7 |

Mean ± SD; identical superscripts (a, b) denote non-significant differences between means in columns (separately for studied elements) according to the post-hoc Tukey’s HSD test

Table S4. Mean concentration [mg kg^-1^] of trace elements with detrimental health effect in soils under particular mushroom species

| Soil | Ag | As | Ba | Cd | Hg | Pb |
| --- | --- | --- | --- | --- | --- | --- |
| So_1_ | 0.048^a^ ± 0.014 | 0.360^b^ ± 0.144 | 14.0^ab^ ±3.54 | 0.201^c^ ± 0.091 | 0.044^b^ ± 0.003 | 9.79^ab^ ± 3.24 |
| So_2_ | 0.042^a^ ± 0.014 | 0.297^b^ ± 0.153 | 15.0^ab^ ± 4.01 | 0.169^c^ ± 0.089 | 0.052^ab^ ± 0.010 | 8.23^b^ ± 1.89 |
| So_3_ | 0.049^a^ ± 0.011 | 0.316^b^ ± 0.128 | 14.9^ab^ ± 3.73 | 0.357^ab^ ± 0.098 | 0.062^a^ ± 0.014 | 11.0^ab^ ± 2.30 |
| So_4_ | 0.034^a^ ± 0.009 | 0.287^b^ ± 0.122 | 12.5^b^ ± 3.67 | 0.248^bc^ ± 0.111 | 0.047^b^ ± 0.006 | 13.7^a^ ± 3.69 |
| So_5_ | 0.035^a^ ± 0.010 | 0.328^b^ ± 0.102 | 18.6^a^ ± 3.55 | 0.195^bc^ ± 0.108 | 0.051^ab^ ± 0.008 | 10.0^ab^ ± 3.24 |
| So_6_ | 0.054^a^ ± 0.013 | 0.533^a^ ± 0.096 | 15.5^ab^ ± 2.78 | 0.465^a^ ± 0.062 | 0.043^b^ ± 0.004 | 10.3^ab^ ± 3.18 |
| So_7_ | 0.043^a^ ± 0.013 | 0.306^b^ ± 0.132 | 14.9^ab^ ± 4.02 | 0.228^bc^ ± 0.100 | 0.052^ab^ ± 0.009 | 11.1^ab^ ± 3.07 |
| Range | 0.020 – 0.078 | 0.090 – 0.682 | 8.51 – 25.7 | 0.018 – 0.542 | 0.039 – 0.085 | 3.95 – 18.8 |

Mean ± SD; identical superscripts (a, b, c) denote non-significant differences between means in columns (separately for studied elements) according to the post-hoc Tukey’s HSD test
